# The Criterion for the Crystallization Ability Assessment as Applied to Borate Glass Powders and Monoliths

**DOI:** 10.3390/e21100994

**Published:** 2019-10-12

**Authors:** Irina G. Polyakova

**Affiliations:** Institute of Silicate Chemistry, Russian Academy of Sciences, nab. Makarova 2, St. Petersburg 199034, Russia; ira_pp@list.ru

**Keywords:** glass stability, crystallization ability coefficient, DTA, glass formation criterion, borates

## Abstract

The glasses of three borate systems, Na_2_O-B_2_O_3_, K_2_O-B_2_O_3_ and BaO-B_2_O_3_, were studied over a wide range of the compositions by differential thermal analysis (DTA) and X-ray powder diffractometry (XRPD). The thermal parameters obtained by DTA method (the glass transition temperature, *T*_g_, the crystallization onset temperature, *T*_x_, and the melting temperature, *T*_m_) were used to calculate the criteria (coefficients) characterizing glass stability against crystallization. The Lu–Liu, Weinberg and Hrubý coefficients were tested for verification of their consistency with several simple requirements. Since each of the criteria has its drawbacks, the coefficient of glass crystallization ability, *K*_cr_, which meets all of the requirements, was also used. The advantage of this coefficient is demonstrated on the example of the glass powders and the monolithic glasses of the mentioned above borate systems.

## 1. Introduction

In many cases, differential thermal analysis (DTA) and differential scanning calorimetry (DSC) allow us in the same experiment to register all of the main characteristic effects accompanying the crystallization of glasses and the subsequent melting [1,2]. The temperatures of these effects can be used to compare coefficients (criteria) that numerically characterize devitrification of glasses. The ease and speed with which such an estimation can be made, enabled researchers to put forward a number of different combinations of characteristic temperatures. Apparently, the first and still most popular coefficient was proposed by Hrubý almost 50 years ago [1]. The 40-th anniversary of the coefficient introduction was marked by a detailed paper of Kozmidis-Petrović & Šesták [3]. Hrubý himself believed that his coefficient, *K*_H_, estimates a glass-forming tendency. He ignored the obvious fact that it was not glass formation, but glass crystallization that occurred during heating in the DTA experiment. This inconsistency was understood and the physical meaning of *K*_H_ and other similar coefficients began to be considered as measure of glass stability (GS) against devitrification. The relationship between GS and the critical cooling rate, which is interpreted as glass forming ability (GFA), was established in a series of publications by Zanotto et al. [2,4,5]. The theoretical analysis of sensitivity for some of the most well-known coefficients was performed in another series of publications by Kozmidis-Petrović [6,7,8]. The author concluded that all of the coefficients are related with GFA but “there is not one that can be declare the best” [8].

In the majority of publications, GS criteria are used to estimate the crystallization resistance for glasses of stoichiometric compositions or for a series of glasses whose composition varies over rather narrow limits. Such a narrow application masks the problems of the coefficients. Here we test some of GS criteria using DTA information for the sodium borate glasses over the whole interval of standard homogeneous glass formation to demonstrate problems resulting from the use of these coefficients. After analyzing the shortcomings of the previously proposed criteria, we introduce the coefficient characterizing the tendency of glasses to crystallize [9,10], trying to avoid the identified shortcomings, and to test this coefficient in the same way that the GS criteria but using three borate system: Na_2_O-B_2_O_3_ and BaO-B_2_O_3_ over the whole range of their standard homogeneous glass formation and potassium borate glasses over the pseudobinary system 5K_2_O·19B_2_O_3_-K_2_O·2B_2_O_3_. The crystallization of the glasses was studied in a dispersed and monolithic state. Crystallization ability of the chosen glasses changes in wide limits: some glasses do not crystalline under the experimental conditions, whilst others do it very easily. The presented work, in which DTA and X-ray powder diffractometry (XRPD) were widely used together to study glass crystallization, inevitably leads to refinement of phase diagrams, which is not the aim of the work and will be mentioned only if necessary. The aim of the work is to build an adequate criterion for assessing the crystallization ability of glass powders and monoliths and to demonstrate the information about the structure of glasses that can be obtained in this way.

## 2. Materials and Methods

Glasses of the sodium borate system were synthesized in Vavilov State Optical Institute in the range of compositions from 0 up to 40 mol.% Na_2_O. Boric acid and Na_2_CO_3_ of ultra-high purity grade were used as raw materials; 100 g of glass of each composition were prepared. The melting temperature varied from 1100 to 1300 °C depending on composition, and the melting time was 2 h. A part of barium borate glasses was synthesized previously for studding their dilatometric properties by the authors of References [11]. Another big part of barium glasses was prepared for this investigation by the same technology: 20–30 g of glasses were synthesized from ultra-high purity grade H_3_BO_3_ and BaCO_3_ during 45 min at 1000–1150 °C. The potassium borate glasses were prepared for the phase diagram investigation [12], and the present author uses these data to estimate their crystallization ability.

DTA was performed in air using a derivatograph (MOM, Mateszalka, Hungary), with the use Pt crucibles and annealed corundum as a reference substance. The heating rate was 10 K/min The glasses were investigated in two states: as fine powders with particle size under 45 μm and in the state of monolithic glass which forms in the crucible after melting the powdered sample. When preparing monoliths, the overheating above the liquidus was 150–200 °C, and the average crucible cooling time from the melt temperature to the glass transition temperature was nearly 2.5 min. The insight into a monolith in this work differs from the occurring in literature designation of pieces of glass with a side of 2–3 mm [13] which we will denote as a bulk glass. Competition between surface and bulk crystallization can manifest itself in such samples, but in glasses poured in the crucible, surface crystallization is weaker, if not completely suppressed. Fire polished surface of the glasses on the boundary with air, if it remains intact, has extremely low crystallization ability as well as the platinum/glass boundary (at least for borate glasses). Monolithic glass in a platinum crucible can be consider as a bulk without surface. In essence, this is the very object to which the notion of glass stability against devitrification should be referred, while fine glass powder can be considered almost as a surface without volume. It is also important that the mass of the monolithic sample is large enough; it should not be a thin film after melting the micro-sample in a micro-crucible. Thin films do not possess a real volume; therefore the crystalline phases formed in them and phase equilibria with their participation may significantly differ from those existing in solid glasses. In our experiments, the weight of the samples was 0.7–0.8 g for sodium and potassium borate glasses and 1.2–1.3 g for barium borate glasses. The number of DTA runs varied usually from one to three for each glass in the powdered state, and from two to five in the monolithic state.

XRPD analysis was used for the identification of crystalline phases. Glass powders crystallized in electric furnaces at temperatures above the temperature of the crystallization peak onset on the DTA heating curves, that is, in the temperature range with a sufficiently high crystal growth rate. In the study of monoliths, the melting point of the crystalline phase sometimes did not match the phase diagram. This usually means that a new compound has formed in the DTA crucible inside the monolithic sample. It is impossible, however, to obtain a monolithic sample for XRPD without destroying the DTA crucible. Therefore, model experiments were performed. Pieces of the glass were placed in a small box made of platinum foil and placed in a DTA furnace on a special holder. Then the experiment was repeated: the model sample was heated, so that it crystallized and then melted, after which it was rapidly cooled to produce solid glass and reheated up to the maximum crystallization temperature of the monolithic glass. When, according to the simultaneously recording DTA curve, the crystallization proceeded intensively, the experiment was stopped, the quenched sample was removed from the substrate and x-ray powder analysis was performed. When the monolith crystallization proceeded slowly or a larger volume of glass was needed, quartz glass crucibles were used. At the boundary between the quartz glass and the borate melt, an interlayer of practically non-crystallizing borosilicate glass is formed. The DTA heating for crystallization and melting for obtaining monolithic glass in a crucible, was simulated in an electric furnace, after which the crucible with the sample in a hot state was transferred to an another furnace preheated to the crystallization temperature. After complete solidification of monolithic glass, the crucible is cooled with it, which leads to its cracking due to the large difference in the thermal expansion coefficients of borate and quartz glasses. After splitting the crucible and removing the transition layer, the resulting polycrystalline sample is used for XRPD analysis.

*Designation of glasses and crystalline compounds.* The glass compositions are labeled as following: the glass 30Na_2_O⋅70B_2_O_3_ is denoted as NB-30, the glass 30K_2_O⋅70B_2_O_3_-KB-30, the glass 30BaO⋅70B_2_O_3_-BaB-30. The stoichiometric compositions are denoted similarly, viz the diborates are indicated as N2B, K2B, and Ba2B. On the phase diagram the stoichiometries are indicated only by numbers, for example, as 1:2 for the diborates.

## 3. Results

*Temperature parameters determined from the DTA curve when heating glasses.* The coefficients that are most often used and will be discussed here [5] are the Hrubý coefficient, *K*_H_, the Weinberg coefficient, *K*_W_, and the Lu–Liu coefficient, *K*_LL_. All these coefficients, similar to many others, are a combination of several temperature parameters determined from the DTA/DSC heating curves. In the literature when determining the temperature parameters, the heating curve of a stoichiometric compound is usually considered. Here we also consider two other cases characteristic for binary systems: the case of a eutectic composition and the common case with a eutectic melting of a mixture of two phases and liquidus dissolution of the primary crystallization phase.

**Figure 1:**
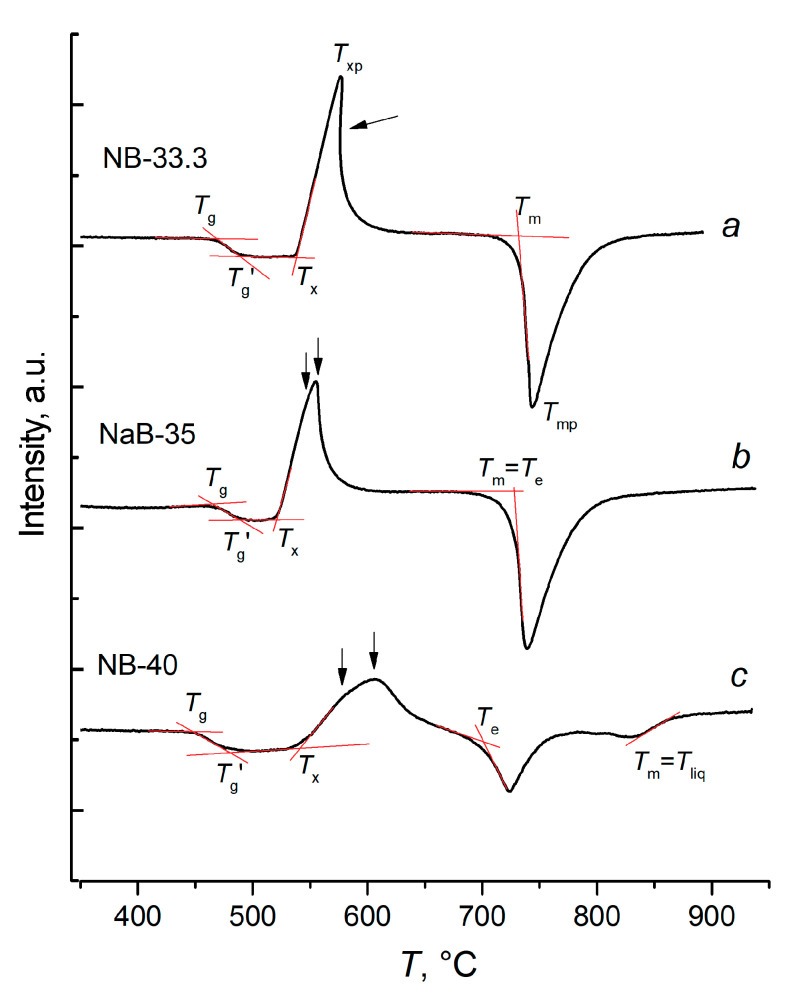
The heating curves of differential thermal analysis for powdered sodium borate glasses with 33.3 (**a**), 35 (**b**) and 40 (**c**) mol.%. Na_2_O.

Let us consider the case of glass of the stoichiometric sodium diborate composition (NB-33.3) in a powdered state (Figure 1a). The first effect in the heating curve of glass is corresponding to the glass transition. It is characterized by the onset temperature (actually *T*_g_) and by the temperature of its end (*T*_g_’), determined by the intersection of the corresponding tangents. Both *T*_g_ and *T*_g_‘ depend on the heating rate and thermal prehistory of the glass and do not depend on its mass. It is generally accepted that in the heating curves crystallization manifests itself after reaching *T*_g_. In fact, the crystallization peak never begins before reaching *T*_g_‘, since a noticeable heat release rate is needed to deviate the baseline. Over the glass transition interval, crystals can nucleate, but their growth rate is negligible [14,15]. The interval *T*_g_–*T*_g_‘ can be considered a dead zone in the study of crystallization by DTA [10].

**Figure 2:**
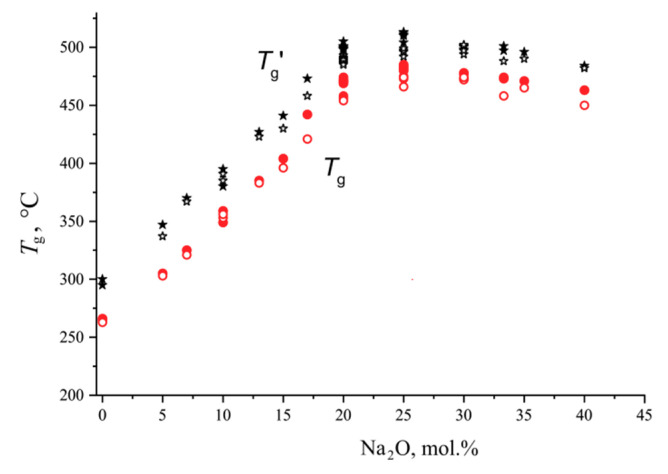
The onset (red) and final (black) glass transition temperatures (*T*_g_ and *T*_g_’, correspondingly) for sodium borate glasses in the powdered (open circles) and monolithic state (filled circles) as a function of composition.

The concentration dependence of *T*_g_ and *T*_g_’ for glasses of the system Na_2_O-B_2_O_3_ is shown in Figure 2. Glass transition temperatures for monoliths coincide or slightly exceed those of powders. The monoliths were formed after crystallization of powders with their subsequent melting. During crystallization of hygroscopic sodium borates, dissolved gases are partially removed, which, apparently, leads to an increase in *T*_g_ of monoliths. The glass transition temperatures of non-hygroscopic barium borate glasses are practically the same for powders and monoliths. The difference between *T*_g_’ and *T*_g_ is approximately 30 °C over the entire composition range.

**Figure 3:**
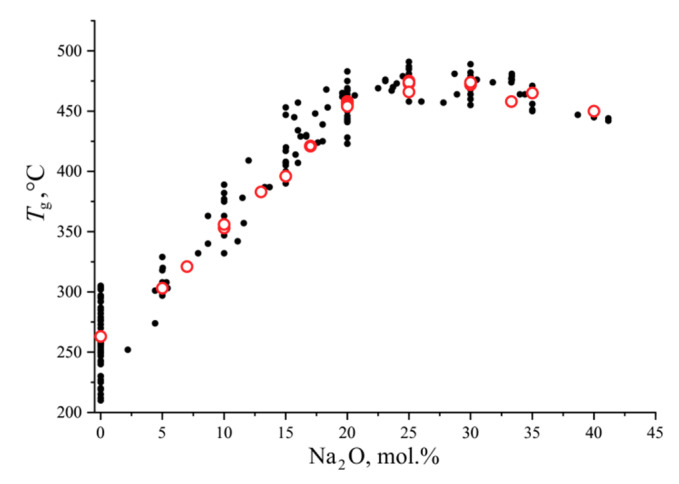
Glass transition temperature, *T*_g_, of sodium borate glasses as a function of composition from this study (red open circles for the powdered samples) and literature [16] (black filled circles).

The glass transition temperatures of sodium borate glass powders are shown in Figure 3 in comparison with the literature data from the SciGlass database [16]. Five large series of measurements on the glasses were used for comparison over the entire studied composition range. For boric anhydride, all data found in Reference [16] used. It can be seen that the data from this study are in good agreement with the measurements reported by other researchers.

The main characteristics of an exothermic crystallization process in the DTA curve is the onset temperature, *T*_x_ (Figure 1a). The maximum temperature of this effect (*T*_xp_) is reached at the maximum conversion rate. In addition, an intensive crystallization under heating may be accompanied by overheating of the sample as a result of thermal autocatalysis. The value of the overheating depends on the heating rate, temperature dependence of the reaction rate and its heat, the mass of the sample, its dispersion, and thermal conductivity conditions. At high crystallization rates and large dispersion of the sample, the overheating can also occur in microcrucibles. In Figure 1, the curve a presents this particular case of the overheating; the arrow indicates a concave part of the heating curve with a negative slope corresponding to the cooling of an overheated sample. Due to high variability, this parameter (*T*_xp_) is rarely used in the construction of evaluative coefficients.

The endothermic melting effect of the crystalline phase is characterized by a single invariant point in the heating curve which is the onset temperature of melting, *T*_m_. The position of this point does not depend on the heating rate and the amount of the sample, but it can decrease with increasing dispersivity due to a strong grinding. The particle size of the powders used in this research is not small enough for detecting this effect. For glass of stoichiometric composition, crystallized by the phase with the sufficient stoichiometry, it is this particular point rather than the extremum of the melting peak that corresponds to the liquidus temperature [17]. The minimum position of the endothermic melting peak, *T*_mp_, strongly depends on the heating rate and the mass of the sample.

The heating curve for the glass of the eutectic composition, NB-35, is shown in Figure 1b. At first glance, it seems to be similar to the heating curve of the stoichiometric glass (Figure 1a). In reality, the two phases, sodium di- and metaborates, crystallize here simultaneously (or almost simultaneously) and simultaneously melt. The onset of the endothermic effect, *T*_e_, corresponds to the eutectic temperature. Criteria defined by a curve of this type refer to the general resistance of glasses towards crystallization (or, conversely, to their crystallization ability), and at the same time to resistance/tendency to the formation of each phase separately. With the simultaneous crystallization of both phases, the coefficients calculated for them are equal.

**Figure 4:**
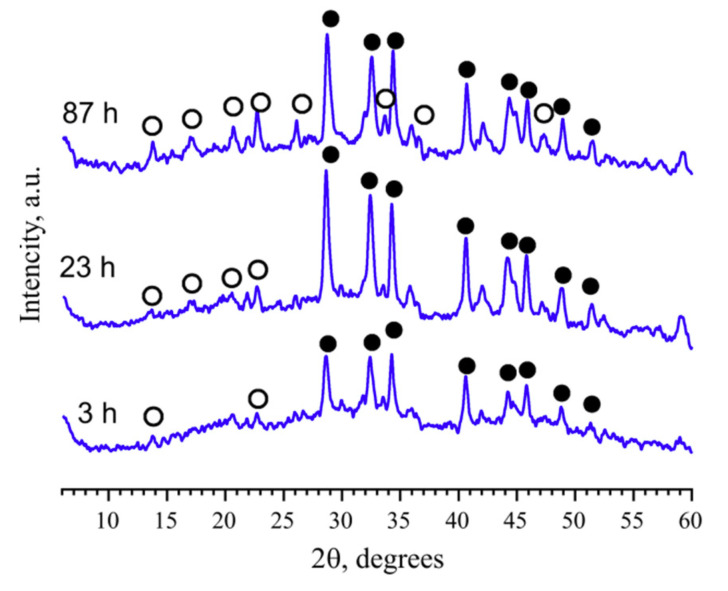
X-ray patterns of the powdered glass NB-40 annealed at 505 °C. The heat treatment time in hours is indicated in the curves. Peaks of the sodium diborate, N2B, are marked by open circles, and that of the sodium metaborate, NB, by filled circles.

A general case of the DTA heating curve for crystallizing two-component glass is shown in Figure 1c. As in the case of a glass of eutectic composition, in the NB-40 glass, di and metaborates (N2B and NB) do not crystallize simultaneously. Two merged maxima, indicated by arrows, are clearly visible in the heating curve at 570–600 °C. The isothermal crystallization of glass powders at 505 °C, which only slightly exceeds *T*_g_’ over the region of low growth rates, allows to determine which of the phases crystallizes first (Figure 4). This is metaborate, NB, whose complete crystallization takes only one day, whilst the crystallization of sodium diborate, N2B, remains far from complete in 3.5 days, as is indicated by the broad maximum of the amorphous scattering. The growth rate of both phases rapidly increases with temperature. At 600 °C, NB-40 glass crystallizes completely in 1 h. Comparison of the x-ray diffraction patterns reveals that, after the heat treatment at 500 °C and during 87 h, only about 30% of the N2B amount crystallizes.

It can be assumed that in the DTA experiment, the metaborate is the first phase to crystallize, and the first maximum in the heating curve (Figure 1c) corresponds to it. Sodium diborate crystallizes later, as the second phase, but when the eutectic temperature is reached, it is completely melted as part of the eutectic mixture with the metaborate. If it is taken into account that in the DTA experiments the sample was heated at 100 °C during 10 min, then the lifetime of the sodium diborate phase would be no more than 15 min.

Sodium metaborate is a primary crystallization phase in the sample NB-40 considered. At the eutectic temperature, two processes proceed: the eutectic mixture, N2B + NB, melts and the liquidus dissolution of the residual sodium metaborate begins. Strictly speaking, the temperatures of both endothermic processes should be considered equal to *T*_e_ which is not true for the liquidus melting metaborate. The extremum temperature of the second exothermic peak in the DTA curve (Figure 1c) is the temperature of the maximum rate of heat absorbtion but, in the case of liquidus dissolution of the primary crystallization phase, this point corresponds to the liquidus temperature and therefore it will be determined further.

As can be seen from Figure 1c, in the glass under consideration, the lifetime of sodium metaborate is approximately 30 min. Obviously, еру resistance of the glass NB-40 to the formation of sodium diborate is higher than to the formation of metaborate. It is possible to use *T*_e_ and *T*_liq_ as *T*_m_ and to determine the stability coefficients separately for each of the phases.

*Error in determining temperature effects.* The accuracy of determining the temperature of effects in the DTA curve is not characterized by the number of decimal places that the built-in processing program can indicate. It is determined by the reproducibility of the results, which is influenced by a set of external and internal factors: the chemical uniformity of the glasses, the identity of the thermal history of different glass fragments, the constancy of the heating rate, etc. To determine the measurement error due to reproducibility, NB-20 glass was chosen as the most difficult object for taking measurements, since the melting effect is preceded by a weak endothermic effect of polymorphic transformation, the processing results of eight experiments being presented in the Table 1. It should be remembered that although average temperatures and the standard deviation can be represented in Celsius, the temperature in calculating the relative error should be indicated in Kelvin.

As can be seen from the Table 1, the onset temperatures of endothermic effects, *T*_g_ and *T*_m_, are best measured. The temperature of crystallization onset is measured with the lowest accuracy. Although the relative error in *T*_x_ measuring remains small, it is twice the error in measuring *T*_g_ and this is by the factor of four larger than the *T*_m_ relative error, which indicates the physical reasons of the *T*_x_ variability.

General requirements for the evaluative coefficients; checking *K*_LL_ and *K*_W_ for the compliance with them by example of Na_2_O-B_2_O_3_ glasses. To compare the glass stability or crystallization ability over a wide range of compositions or for different systems, it is necessary to be sure that the criteria used are comparable. There are formal conditions that must be met by a coefficient that evaluates the degree of implementation of the process. The coefficient should be:

1. dimensionless,
2. bonded (it should not become infinite at the singular points),
3. normalized (vary from zero to one),
4. linear.

Let us check the Lu–Liu coefficient, *K*_LL_, the Weinberg coefficient, *K*_W_, and the coefficient of Hrubý, *K*_H_ for compliance with these conditions. When we investigate any glass by DTA, *T*_g_ and *T*_m_ are the fixed parameters and only *T*_x_ can change, e.g., when the dispersion or heating rate changes. Thus, the temperature *T*_x_ is a variable in the equations for the coefficients and it is easily to see that *K*_LL_ and *K*_W_ depend on it linearly:

(1) $$K_{\mathrm{LL}}=\;\frac{T_{x}}{\left(T_{g}+T_{m}\right)},$$

(2) $$K_{W}=\;\frac{\left(T_{x}-T_{g}\right)}{T_{m}}.$$

**Figure 5:**
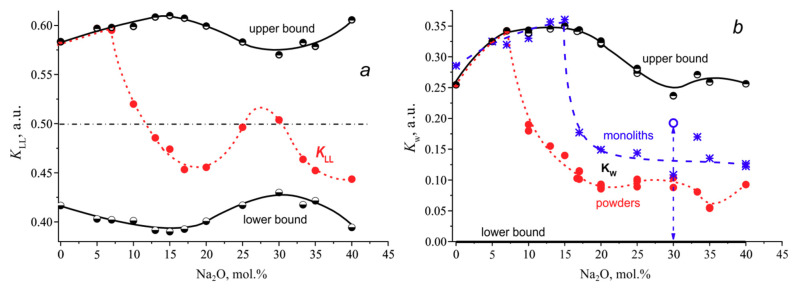
Glass stability criteria *K*_LL_ (**a**) and *K*_W_ (**b**) for glasses of the system Na_2_O-B_2_O_3_. The filled red circles are the coefficients for powdered glasses; the half-filled circles are bounds of the coefficient definition areas for them. The bleu asterisks (**b**) are *K*_W_ for monoliths; the blue open circle at 30 mol.% Na_2_O is the upper bound for the monolithic glass.

Both coefficients are dimensionless and limited in magnitude, but the bounds of the region of existence of the coefficients depend on the composition. *T*_x_ can vary from *T*_g_ (in fact, from *T*_g_’) to *T*_m_, and accordingly *K*_LL_ can vary from *T*_g_/(*T*_g_ + *T*_m_) to *T*_m_/(*T*_g_ + *T*_m_) and *K*_W_- from zero to (*T*_m_ − *T*_g_)/*T*_m_. Let us consider by example of the sodium borate system how these coefficients behave in the composition range from 0 to 40 mol.% Na_2_O (Figure 5). The filled red points and dashed lines represent changes in the *K*_LL_ and *K*_W_ values with composition for glass powders. The half-filled circles and solid lines show the position of the upper and lower boundaries of the coefficient existence region: they can vary between the boundaries but cannot go beyond them.

**Figure 6:**
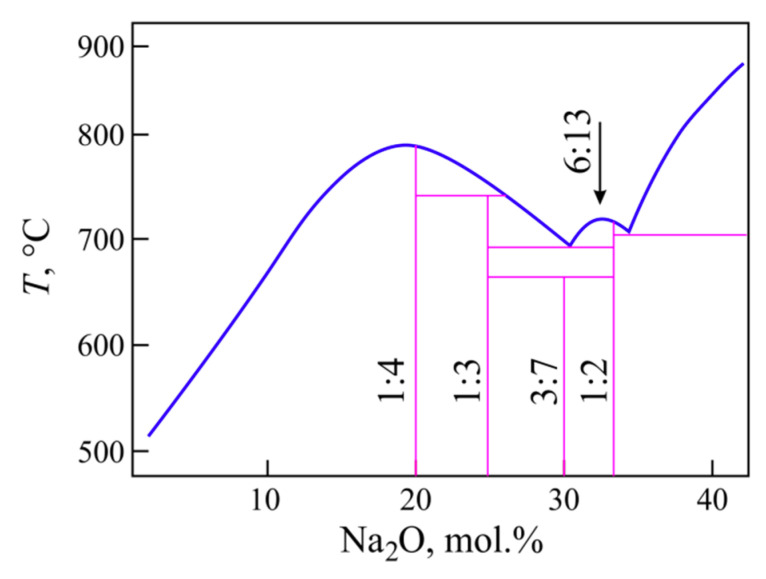
Summary scheme of the Na_2_O-B_2_O_3_ phase diagram over the glass formation region.

As can be seen from Figure 5a, the *K*_LL_ definition area, its upper and lower bounds are symmetric with respect to the horizontal axis *K*_LL_ = 0.5. The shape of the bounds is determined by the concentration dependence of *T*_g_ and the shape of the liquidus line in the phase diagram of the Na_2_O-B_2_O_3_ system. The phase diagram of the sodium borate system is well known; it is presented schematically in Figure 6 based on the diagram of [18] supplemented with the 3N7B and 6N13B compounds. The first of these compounds appears in [19] as 2N5B, but the definition of the crystal structure in [20] determines its stoichiometry as 3Na_2_O·7B_2_O_3_. The crystal structure of the second compound, 6Na_2_O·13B_2_O_3_, was established in [21]. A little earlier, its liquidus was constructed by Kaplun & Meshalkin [22].

From zero to 20 mol.% Na_2_O, the liquidus temperatures change by 350 °С, and the glass transition temperature by 250 °C. When the Na_2_O content is in the range from 20 to 40 mol.%, the change in the liquidus temperature is less but it passes through two eutectics at 30 and 35 mol.% Na_2_O (Figure 6), and besides the second eutectic is weakly pronounced. Since the liquidus is known, the bounds can also be calculated for non-crystallizing glasses. For data consistency it is better, first, to crystallize the powders of such glasses and then to determine *T*_m_ in a separate DTA experiment. Since B_2_O_3_ does not crystallized under ordinary conditions (the maximum crystallization resistance), the value for its *T*_m_ is taken from the literature. Calculated in this way, both bounds of the *K*_LL_ area of existence (Figure 5a) have a complex shape reflecting the maxima and eutectic minima of the liquidus. The red line depicting *K*_LL_ shows that the addition of Na_2_O to boric anhydride first slightly increases the stability of the glass, which then sharply decreases reaching a minimum near the tetraborate composition, N4B. A further increase in the content of sodium oxide in the glasses leads to an increase in the value of *K*_LL_ (i.e., the stability of the glasses) in the vicinity of the eutectics and its further decrease when approaching the glass formation boundary. In general, a change in the *K*_LL_ value with composition qualitatively correctly reflects a change in the stability of glasses with the exception of the initial range, 0–7 mol.% Na_2_O. The coincidence of *K*_LL_ for these glasses with the upper bound means that these glasses do not crystallize. If the bounds of the area of existence for *K*_LL_ are not constructed, it is impossible to draw such a conclusion only from the run of the curve showing the concentration dependence of *K*_LL_. Moreover, the initial part of the curve *K*_LL_ gives qualitatively incorrect information about the change in the stability of glasses with composition. It is known that boric anhydride cannot be crystallized without special techniques, and therefore its resistance to crystallization is maximum. It is the addition of a modifier cation that allows the borate glass to crystallize.

Problems may arise with comparing the coefficients for crystallizing glasses, too. Can it be said that the same *K*_LL_ values, for example, for NB-17 and NB-35 (Figure 5a), do reflect the same stability of the glasses? Obviously not. The first composition lies in the region of the maximum width of the *K*_LL_ existence area, whilst the second is in its narrowest part. The *K*_LL_ values should be measured off from its lower bound, and this interval should correlate with the size of the allowed zone for the composition considered. When using *K*_LL_, one can speak about the similarity in the stability of glasses only if *K*_LL_ = 0.5 or when considering glasses from a narrow composition range, where the width of the region of existence of the coefficient changes insignificantly.

The use of *K*_W_ for an estimation the stability of glasses against crystallization leads to the similar problems (Figure 5b). The red filled dots and the dashed line represent the concentration dependence of *K*_W_ for sodium borate glass powders. The lower bound of the *K*_W_ existence area is a straight line coinciding with the abscissa axis. All of the liquidus features are located in the upper bound profile. Anyone who, without knowing the shape of the bounds, makes such a conclusion will be mistaken. The concentration dependence of *K*_W_ in the range from 17 to 30 mol.% Na_2_O reveals that the glasses are equally stable against crystallization. The region of *K*_W_ existence sharply narrows in the above composition interval with an increase in the Na_2_O content, and its fraction realized in the coefficient increases as it approaches the eutectic composition of NB-30.

As in the case of *K*_LL_, at low contents of sodium oxide, the concentration dependence of *K*_W_ coincides with the upper boundary, since at the used heating rate of 10 K/min, crystallization of glass powders occurs only if the Na_2_O content in the glass is 10 mol.% or more. As already mentioned, glasses with a lower content of Na_2_O do not crystallize during heating and their relative stability cannot be compared but the *K*_W_ dependence shows significant rise in glass stability with Na_2_O addition to B_2_O_3_. This problem becomes even more obvious when using the monolithic glasses (the blue asterisks and dashed line in Figure 5b). Monoliths begin to crystallize only when the Na_2_O content in the glass reaches 17 mol.%. The stability of the glasses with a lower Na_2_O content cannot be estimated numerically because the glasses do not crystallize. Nevertheless, *K*_W_ shows that the NB-15 glass has the maximum stability over the whole glass formation range, and in particular it is 20% higher than that of boric anhydride, which is certainly wrong.

**Figure 7:**
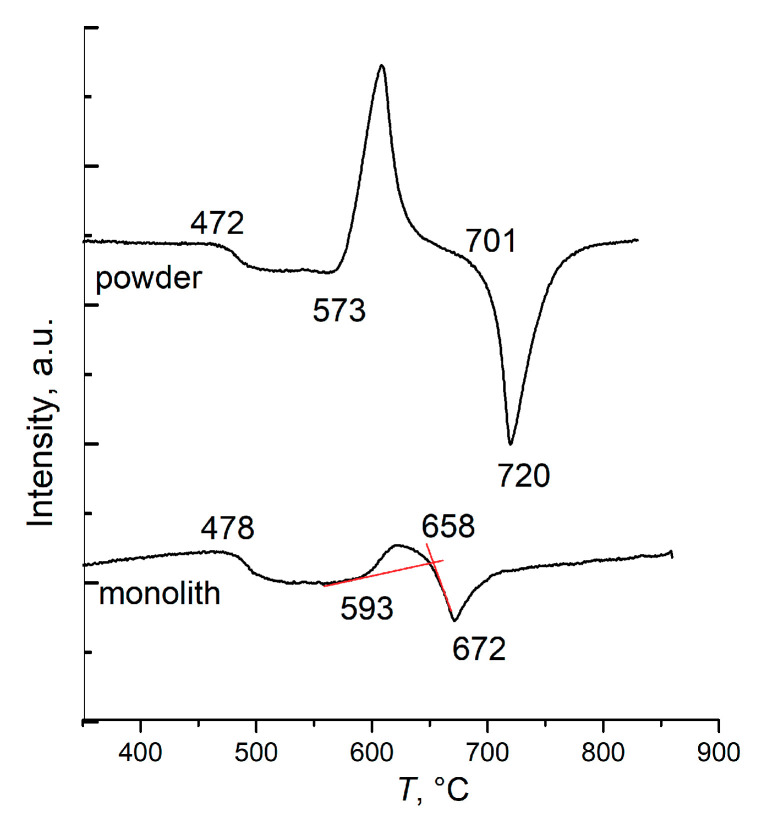
Heating curves of the powdered and monolithic NB-30 glass.

The small difference in the *K*_W_ upper bounds for powders and monoliths in the small alkaline region is due to the natural spread of data or a possible small difference in the glass transition temperatures for the powders and monolithic glasses. The upper *K*_W_ bound for monoliths (it is not completely shown in Figure 5b so to avoid cluttering up the figure) is close to the upper bound for glass powders, with the exception of one point for NB-30 glass. Glasses of the eutectic composition can crystallize differently in the powdered and monolithic state during the DTA experiments. The DTA curves in Figure 7 represent such a case for NB-30 glass. The heating of the powder leads to simultaneous crystallization of the eutectic mixture N3B + N2B and to subsequent simultaneous eutectic melting at *T*_m_ = 701 °С. When the monolithic glass is heated, the compound 3N7B crystallizes, which melts at *T*_m_ = 658 °C. Thus, for the glass NB-30 the DTA experiment gives different liquidus temperatures for the samples of different dispersion. The open circle in Figure 5b shows the position of the *K*_W_ upper bound for the monolithic glass under consideration. It lies noticeably below the bound for the glass powders. As a result, although *K*_W_ for the powder and monolith of NB-30 glass coincide (Figure 5b), their stability cannot be considered as equal, since the regions of existence for this glass in powder and monolith are different. Of course, the coefficient *K*_LL_ for powder and monolith of NB-30 glass demonstrates a seemingly equal stability.

Thus, the lack of normalization limits the applicability of the coefficients *K*_LL_ and *K*_W_ by narrow ranges of compositions. When used over a wide range of compositions, the use of these coefficients can lead to the incorrect conclusions.

*Testing the Hrubý coefficient.* The Hrubý coefficient characterizing the glass stability against crystallization contains the variable *T*_x_ in both the numerator and the denominator, but can easily be converted to the form of a power function:

(3) $$K_{H}=\;\frac{\left(T_{x}-T_{g}\right)}{\left(T_{m}-T_{x}\right)}=\;\frac{\left(T_{m}-T_{g}\right)}{\left(T_{m}-T_{x}\right)}-1.$$

**Figure 8:**
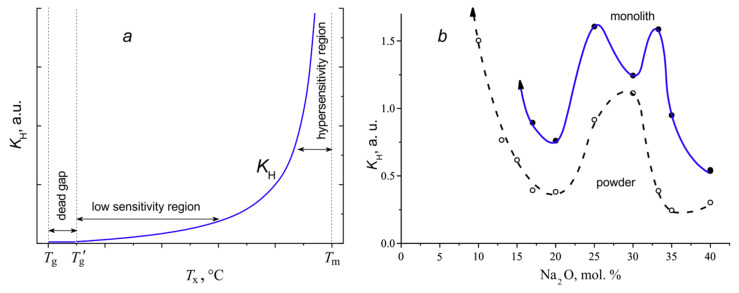
The Hrubý coefficient, *K*_H_: (**a**) the *T*_x_ dependence at fixed *T*_g_ and *T*_m_; (**b**) the composition dependence for sodium borate glasses in the powdered and monolithic states.

For a glass of a given composition with the fixed temperatures *T*_g_ and *T*_m_, the value of *K*_H_ varies from zero at *T*_x_ = *T*_g_ to infinity at *T*_x_ = *T*_m_ (Figure 8a). As already noted, the temperature range from *T*_g_ to *T*_g_’ is a dead zone, since the crystallization cannot begin in this region in the DTA experiments. At the temperatures above *T*_g_’, there is a rather extended region of the low sensitivity of the coefficient. When the *T*_x_ value approaches to *T*_m_, the *K*_H_ sensitivity with respect to changes in *T*_x_ rapidly increases (the hypersensitivity region). The same change in the value of *T*_x_ leads to significant differences in the changes in *K*_H_ in the regions of low and high sensitivity of the coefficient. Obviously, the coefficient is of little use for a quantitative comparison.

Figure 8b represents the concentration dependence of the *K*_H_ coefficient for the sodium borate glasses in the powdered and monolithic states. The shape of these dependences resembles that for the dependences for *K*_LL_ and *K*_W_. The figure shows that glass powders are less resistant to crystallization than monoliths. The maximum of the liquidus curve in the phase diagram (Figure 6) for the NB-20 composition is also reflected in the dependence of the *K*_H_ coefficient in the form of a minimum. The eutectics at NB-30 manifests itself as the maximum stability for powders, but as the minimum for monoliths due to the formation of the compound 3H7B. However, with a decrease in the sodium oxide content, the *K*_H_ concentration dependences for powders and monoliths go to infinity and cannot be shown in the graph.

Thus, the Hrubý coefficient is dimensionless, but it is non-linear, unlimited at the singular point at *T*_m_ and, as a consequence, is not normalized. The previous consideration showed that, although the Lu–Liu and Weinberg coefficients are dimensionless, linear and limited, the lack of normalization prevents their correct quantitative use over wide composition intervals or for comparing the stability of glasses of different systems. One of the papers by Kozmidis–Petrović [8] devoted to the assessment of the stability criteria for glasses on the basis of DTA experiments is entitled: “Which glass stability criterion is the best?” Apparently, the answer should be that all of them are not good enough.

*The proper definition of the coefficients satisfying the above requirements.* The coefficient satisfying all of the above conditions should reflect the difference between the thermal parameters in the numerator and denominator. The parameter *T*_x_ should be included only in the numerator, and the denominator should contain the term (*T*_m_ − *T*_g_), that is, the length of the temperature interval in which crystallization of the glass can occur. Two coefficients can be defined in this way. The first is the coefficient of the glass stability against crystallization.

(4) $$K_{\mathrm{GS}}=\;\frac{\left(T_{x}-{T_{g}}^{\prime }\right)}{\left(T_{m}-{T_{g}}^{\prime }\right)}.$$

The value of the *K*_GS_ coefficient varies linearly from 0 for glasses which crystallize immediately after the end of the glass transition effect during the DTA experiment (*T*_x_ = *T*_g_’) and up to 1 in the case of non-crystallizing glass (*T*_x_ = *T*_m_). The temperature of the end of the glass transition effect, *T*_g_’, is used instead of *T*_g_ to exclude the needless dead zone (Figure 8a) from a consideration.

The second coefficient seems to be more logical when the DTA experiments are used. The coefficient of glass crystallization ability was first introduced in [9] for K_2_O-B_2_O_3_ glasses and used later to describe crystallization in the BaO-B_2_O_3_ system [10]:

(5) $$K_{\mathrm{cr}}=\;\frac{\left(T_{m}-T_{x}\right)}{\left(T_{m}-{T_{g}}^{\prime }\right)}.$$

The temperature *T*_g_’ instead of *T*_g_ is also used to exclude the dead zone from a consideration. The *K*_cr_ coefficient varies from 1 to 0 with a change in the temperature of the crystallization onset, *T*_x_, from *T*_g_ to *T*_m_. Thereby, *K*_cr_ = 0 for non-crystallizing glasses and *K*_cr_ = 1 for glasses which crystallize immediately upon a completion of the glass transition effect in the DTA process.

Several examples of the use of *K*_cr_ for characterizing the crystallization of glasses in different borate systems are given below. It should be remembered that the temperature parameters determined from the DTA curves are not universal but depend on the experimental conditions, in particular, on the heating rate and dispersion of the sample. In this paper, all of the examples of calculating the coefficients relate to the heating rate of 10 K / min, and the dispersion of the samples is specified in each case.

**Figure 9:**
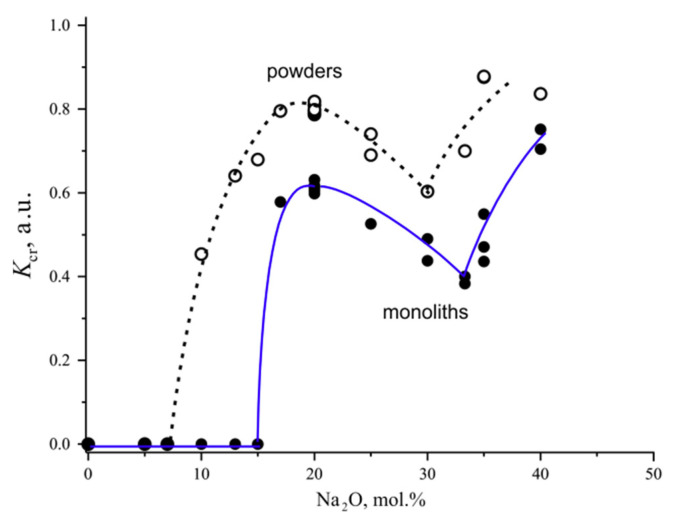
The composition dependence of the coefficient of crystallization ability, *K*_cr_, for the powdered and monolithic sodium borate glasses.

*The crystallization ability coefficient for Na_2_O-B_2_O_3_ glasses.* The *K*_cr_ coefficient for sodium borate glasses is presented in Figure 9. At the low Na_2_O content *K*_cr_ = 0 which means that the glasses did not crystallize over this composition region; this is evident from the appearance of the graphs. With an increase in the Na_2_O content, the crystallization ability of the powders gradually increases starting from 10 mol.%. The crystallization ability of the monoliths, expressed by *K*_cr_, increases abruptly from zero to an almost peak value upon the composition change from NB-15 to NB-17. The *K*_cr_ maximum of both the powdered and monolithic glasses is located at the tetroborate composition, N4B, containing 20 mol.% Na_2_O. The eutectic with the NB-30 composition, which was already discussed, is clearly manifested as a minimum in the dependence of *K*_cr_ for powders. For monoliths, however, a minimum of the crystallization ability is observed at the stoichiometric composition of sodium diborate, NB-33.3. Perhaps this is due to the existence in the system of a compound close in composition to the diborate, with the complex stoichiometry 6N13B (Figure 6, [21]). A further increase in the Na_2_O content and approaching the boundary of the glass formation region leads to an increase in the *K*_cr_ coefficient of both the powders and monoliths.

**Figure 10:**
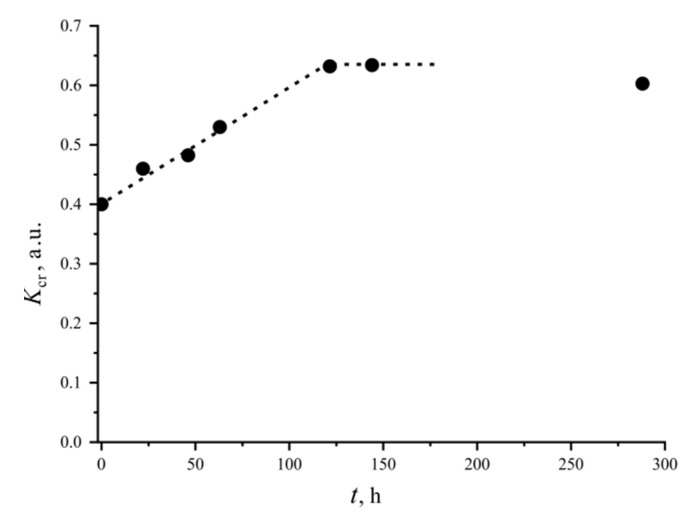
The crystallization ability coefficient, *K*_cr_, for the monolithic NB-33.3 glass depending on the heat treatment time at 500 °C.

Since the coefficient satisfies all of the above requirements, it is acceptable to quantify the difference in crystallization ability of powders and glass monoliths. Thus, at a maximum at NB-20, the value of the *K*_cr_ coefficient for the powders is 1.3 times greater than it is for the monoliths. Another example of the quantitative use of the proposed criterion is presented in Figure 10. Platinum crucibles with NB-33.3 monolithic glass were heat-treated at a temperature of 500 °C, which is slightly higher than the *T*_g_ of this glass and is close to *T*_g_’. After exposure, the DTA experiment was performed and the *K*_cr_ coefficient was calculated from the found temperature parameters. After the heat treatment, the glasses remained transparent. However, as Figure 10 shows, *K*_cr_ increases linearly with the exposure time due to a shift of *T*_x_ towards a lower temperature. Apparently, bulk nucleation of sodium diborate occurred in the monolithic glass. With an increase in the heat treatment time to 5 days or more, the crystal growth began to appear. The samples began to turn white, and the coefficient stopped changing. After 14 days, the monolith completely crystallized. A discussion of the features of the nucleation and growth of the N2B crystals in the monolithic glass is beyond the scope of this paper. However, it is obvious that the *K*_cr_ coefficient is suitable for indicating the bulk nucleation of crystals in glasses.

**Figure 11:**
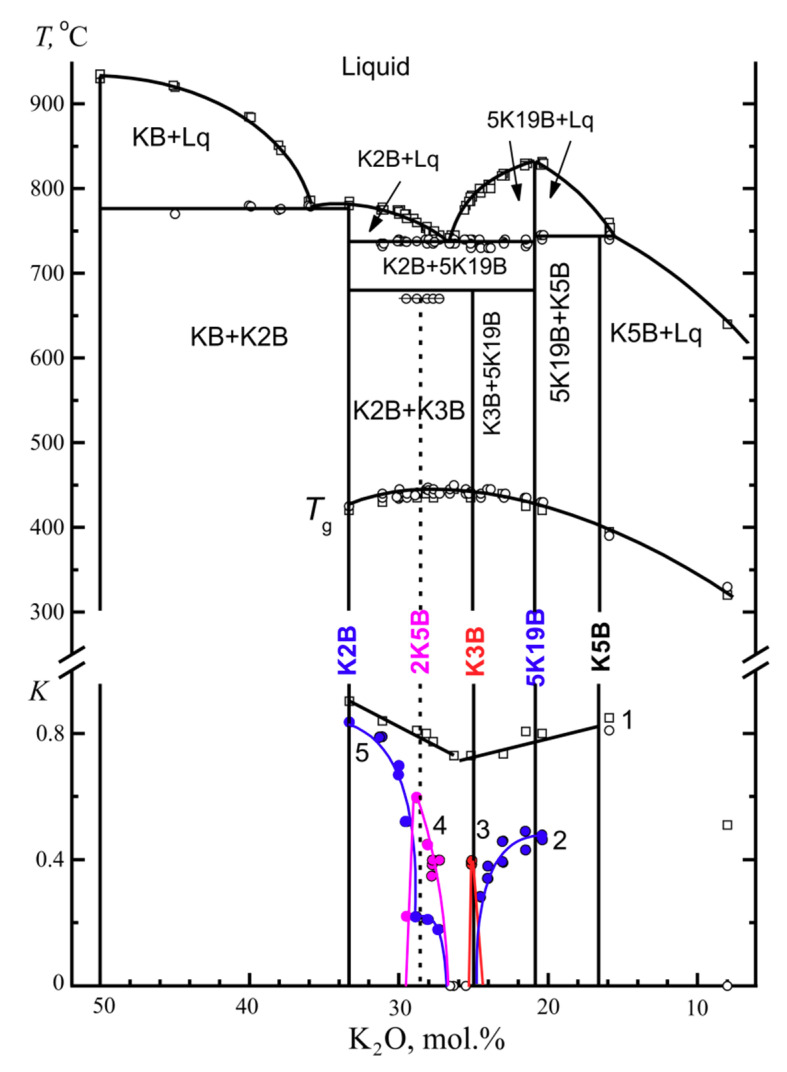
The phase diagram of the K_2_O-B_2_O_3_ system according to [12] (above), the glass transition temperatures, *T*_g_ (in the center), and the crystallization ability coefficient, *K*_cr_, for the potassium borate glasses (below) in the powdered (open squares) and monolithic state (filled circles). The crystalline phases to which the *K*_cr_ coefficient relates are K2B+5K19B (1), 5K19B (2), K3B (3), 2K5B (4), and K2B (5).

*The separate determination of K_cr_ for the individual crystalline phases in the K_2_O-B_2_O_3_ system.* The phase diagram of the particular binary system K_2_O⋅2B_2_O_3_-5K_2_O⋅19B_2_O_3_ (Figure 11) was studied and described in detail in [12]. To identify the processes leading to the appearance of thermal effects in the DTA curves, a simulation of the processes in a DTA furnace was used in which crystallization and fusion of the samples were performed with quenching at different stages of the development of the processes and with a subsequent XRPD analysis for the phase identification.

**Figure 12:**
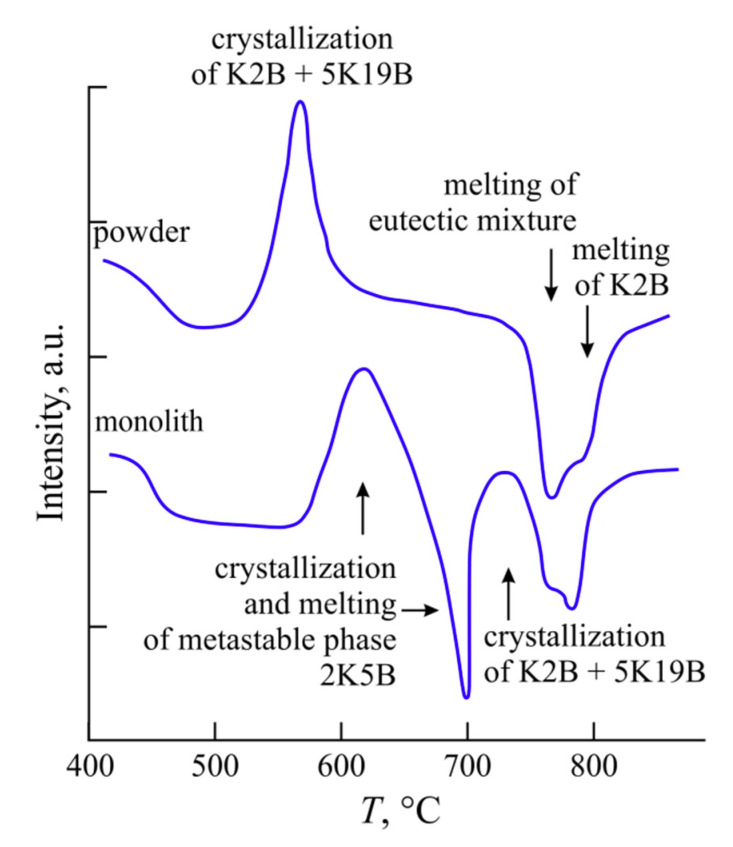
The DTA curves representing the crystallization and melting of the KB-28 glass in the powdered (above) and monolithic (below) state.

It was shown that two bulk crystallizing phases form in the K_2_O⋅2B_2_O_3_-5K_2_O⋅19B_2_O_3_ system near the eutectic composition. The specifics of the K3B crystallization are described in [12]. The x-ray diffraction data of the crystalline phase 2K5B are given in [9]. This compound only forms in the monolithic samples. The DTA curves in Figure 12 show how crystallization and melting occur in a powder and a monolith of the glass, which is close in the composition to the 2:5 stoichiometry. In the glass powder, the mixture of K2B + 5K19B crystallizes and melts in accordance with the equilibrium phase diagram. In the monolith, the 2B:5K compound first crystallizes and melts at 680 °C. The mixture of K2B and 5K19B begins to immediately crystallize in the resulting melt and then the crystals that formed melt in turn, in accordance with the equilibrium phase diagram. The temperature parameters *T*_g_’, *T*_x_, and *T*_m_ can be determined from the DTA curve for the monolithic KB-28 glass (Figure 12) separately for each of the crystalline phases, K2B, 2K5B, and 5K19B. The *T*_g_’ value is naturally the same for all of the phases, but the *T*_x_ and *T*_m_ values are different. Moreover, for the K2B and 5K19B phases, the crystallization onset temperature almost coincides with the minimum temperature in the 2K5B melting curve. This is evidenced by almost vertical trailing edge of this endothermic effect. After completion of the endothermic process, the return of the DTA signal to the baseline occurs more slowly. This can be seen, for example, from the trailing edge of the K2B melting effect in the same DTA curve. In this case, after 2K5B melting, the return of the signal is accelerated by the released heat of crystallization of the two equilibrium phases.

The crystallization ability coefficient, *K*_cr_, for the potassium borate glasses is presented in Figure 11, below. The points and line 1 represent the concentration dependence of *K*_cr_ for glass powders, and the liquidus temperatures were used as *T*_m_ in the calculation. Consequently, this line represents a tendency toward crystallization of the primary crystallization phases. To the left of the eutectic, there is the K2B phase, to the right-5K19B, and the coefficient can be interpreted as that referring to these phases. At the same time, the line 1 characterizes the general ability of the powdered glasses to crystallize. The line 1 has a clear minimum in the composition of the eutectic.

The points and lines 2 to 5 in Figure 11, below, characterize the tendency of the individual phases towards crystallize in the monolithic glasses. The *K*_cr_ values for the primary crystallization phases 5K19B (the line 2) and K2B (the line 5) sharply decrease and drop to zero as the glass compositions approach the eutectic composition. Two sharp peaks of the *K*_cr_ dependencies for volume-crystallizing phases K3B (the line 3) and 2K5B (the line 4) are located closely to the right and to the left of the region of glasses that do not crystallize in a monolithic state. The crystallization region of the potassium triborate, K3B, is extremely narrow, and a deviation from the stoichiometric composition, KB-25, by 0.5 mol.% leads to a complete cessation of crystallization of this compound. The crystallization region of the 2K5B compound is noticeably wider reaching 1.7 mol.%, and the *K*_cr_ coefficient at the maximum value is one and a half times greater than *K*_cr_ in the case of the potassium triborate. On the line 5 for K2B, the shoulder with *K*_cr_ = 0.2 within the 2K5B crystallization region can be explained by crystallization of the potassium diborate that occurs already after melting of the metastable 2K5B compound, as shown in the DTA curve for the KB-28 monolith in Figure 12.

*The crystallization ability coefficient for the BaO-B_2_O_3_ system.* The phase diagram of the system was quite well studied [23,24,25], especially after outstanding nonlinear optical properties were discovered for β-BaO⋅B_2_O_3_ (the review in Reference [26]. Over the composition range studied, the 4Ba7B compound [27] and the low-temperature volume-crystallizing modification of barium diborate, β-Ba2B [28], were found.

**Figure 13:**
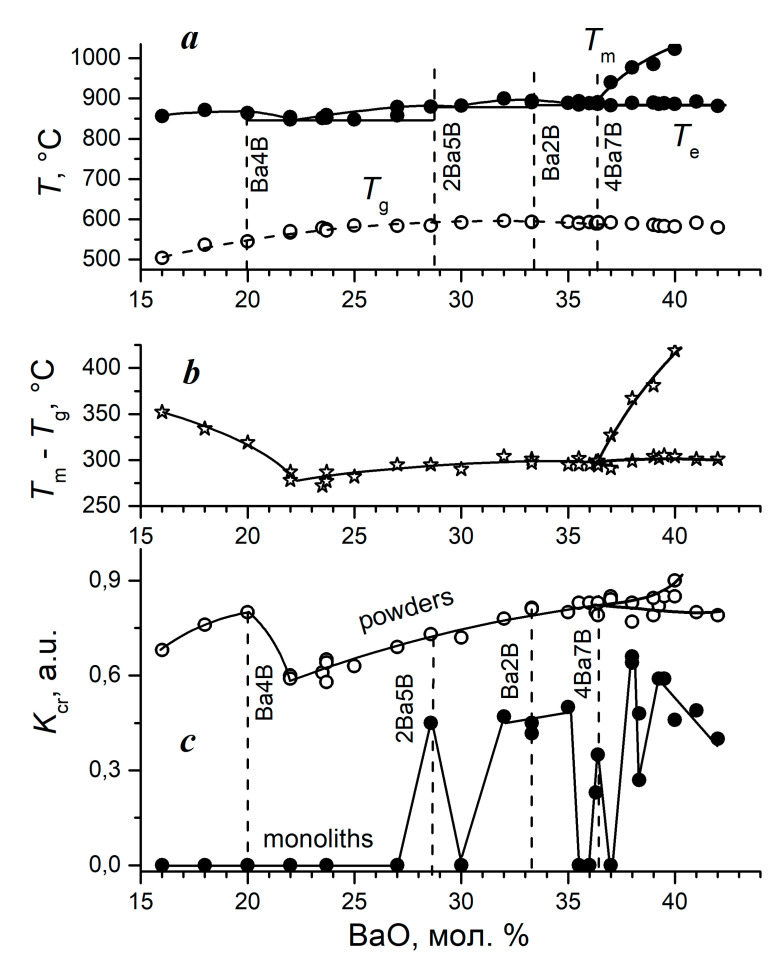
Concentration dependences for the barium borate system according to DTA: (**a**) the melting temperature, *T*_m_ (the filled circles), and the glass transition temperature, *T*_g_ (the open circles); (**b**) the difference (*T*_m_ − *T*_g_); (**c**) the crystallization ability coefficient, *K*_cr_, for the glass powders (the open circles) and for the monolithic glasses (the filled circles).

In the barium borate system, the region of homogeneous glass formation begins at about 16 mol.% BaO. At the lower content of barium oxide, the stable liquid-liquid phase separation occurs in glasses; and it is impossible to quench them in a single phase state. Therefore, only glasses containing from 16 to 43 mol.% BaO were studied. The results of the DTA investigation of the BaO-B_2_O_3_ system, partially published in [10], are presented in Figure 13. A specific feature of this diagram is an extended interval with a very gentle liquidus line. A specific feature of this diagram is an extended region with a very flat liquidus line. The changes in the *T*_m_ values do not exceed 60 °C over the range from 16 to 36.6 mol.% BaO (Figure 13a). Three eutectics are located in this interval: between the Ba4B-2Ba5B, 2Ba5B-Ba2B, and Ba2B-BaB compounds. From the BaB-36.6 composition towards a higher content of barium oxide, the primary crystallization field of barium metaborate begins and is bounded below by the eutectic horizontal *T*_e_.

It is usually assumed that crystallization during heating at a constant rate is the more likely the greater is the (*T*_m_ − *T*_g_) temperature interval in which it can take place. This interval is presented in Figure 13b. It has a minimum at BaB-22 (the composition of the first eutectic), and a kink at BaB-36.6 (the composition of the third eutectic). The second eutectic, whose composition is that of BaB-30 does not manifest itself in any way in this dependence.

The concentration dependence of the crystallization ability coefficient for the barium borate glass powders (Figure 13c) has a maximum at BaB-20, i.e., at barium tetraborate composition. The dependence of *K*_cr_ has a pronounced minimum at the eutectic composition. The increase in the BaO content is accompanied by a slow increase in *K*_cr_, without any features at the compositions of other eutectics. In general, the concentration dependence of *K*_cr_ for powders reflects the features of the concentration dependence of (*T*_m_ − *T*_g_). In this case, a change in the crystalline phases with a change in the composition does not affect the shape of the concentration dependence of *K*_cr_, except for in the vicinity of the BaB-20 composition.

The *K*_cr_ coefficient for monoliths (Figure 13c) varies completely differently depending on the composition. The monolithic barium borate glasses do not crystallize over the composition range from 16 to 27 mol.% BaO, *K*_cr_ = 0. With an increase in the BaO content, the coefficient value reaches 0.5 for the glass of the stoichiometric composition 2Ba5B (BaB-28.6) and immediately drops to zero for the glass with the eutectic composition BaB-30. The coefficient has a rather wide maximum near the composition of barium diborate, and the β-modification of the diborate [28] crystallizes here, while α-Ba2B forms in the glass powders of the same compositions. With a change in the composition from BaB-35 to BaB-35.5, *K*_cr_ drops sharply to zero again. Then, upon reaching the eutectic composition, the coefficient sharply increases as a result of the formation of a compound with an almost eutectic composition, 4Ba7B [27] and it turns to zero for BaB-37 glass.

With a further increase in the BaO content, several more crystalline phases form in glasses both during the DTA heating and in isothermal experiments. These compounds and their mutual transformations are not sufficiently studied by now. The concentration dependence of *K*_cr_ over the range from 38 to 42 mol.% BaO cannot be reliably interpreted at present. However, the following fact is noteworthy. The coefficient value at the composition of BaB-38 reaches its maximum for monolithic barium borate glasses and then begins to decrease when approaching the glass formation boundary. Perhaps, this decrease in *K*_cr_ for the monoliths explains the strange fact that glasses BaB-41 and BaB-42 glasses are easier to obtain by cooling in a crucible than by traditional quenching on a plate.

If the ability of monolithic barium borate glasses to resist crystallization were represented as the Hrubý coefficient, *K*_H_, the most part of the graph would go to infinity.

## 4. Discussion

Does it make sense to calculate coefficients like *K*_cr_? After performing DTA of glasses, the experimenter already knows which glasses crystallize easily, which crystallize with difficulty, and which do not crystallize at all. Will they gain a new knowledge after plotting graphs like the ones presented above? Apparently, yes. Firstly, a properly defined coefficient really allows us to compare. Apparently yes. Firstly, the correctly determined coefficient allows us to compare the crystallization ability of glasses not only within the same system, but also for different systems, as well. Secondly, the construction of concentration dependences of *K*_cr_ allows us also to present a large and complex body of experimental data in a visual and compact form.

**Figure 14:**
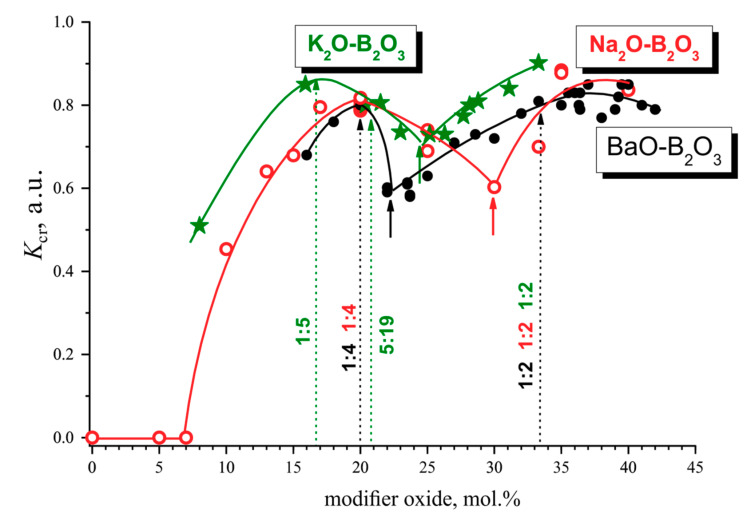
Comparison of the composition dependence of the crystallization ability coefficients, *K*_cr_, for glass powders of the systems: Na_2_O-B_2_O_3_ (the red open circles); K_2_O-B_2_O_3_ (the green asterisks), and BaO-B_2_O_3_ (the filled black circles).

Figure 14 represents such a comparison for the glass powders of three studied borate systems, sodium, potassium and barium. Over the region of compositions from 15 to 42 mol.% of the modifying oxides, the *K*_cr_ values for all of three systems lie within the interval 0.6 < *K*_cr_ < 0.9. Nevertheless, the crystallization ability of potassium borate glass powders is slightly higher than that of the sodium and barium borates. Over the region from 7 to 20 mol.%, the dependences of *K*_cr_ have approximately the same slope. The difference in the *K*_cr_ values near the eutectic compositions and in the nearest maximum does not exceed 0.2. In all of the systems studied, a minimum in the concentration dependence of *K*_cr_ is observed for eutectic compositions (short arrows in the figure) closest to the first maximum in the B_2_O_3_ rich region. It can also be said with confidence that the *K*_cr_ value in the BaO-B_2_O_3_ system monotonically increases in the composition range from 22 to 38 mol.% BaO. The *K*_cr_ values belong to the line, which is common for a very long range of compositions. The presence of the eutectics and the repeated change of crystallizing phases do not matter here. Apparently, for the crystallization ability of glass powders, a decrease in the connectivity of the anionic framework with an increase in the content of modifier oxide plays a decisive role.

The number of glass compositions studied in the sodium borate system is quite sufficient to investigate the usual properties of glasses. Unfortunately, it is completely insufficient to study the phase diagram. The coefficient of the crystallization ability of glasses, especially in the monolithic state, shows pronounced changes with small changes in the glass composition, especially in the vicinity of eutectics (Figure 11 and Figure 13). To study similar variations in *K*_cr_ for the sodium borate glasses, it is necessary to study a large number of glasses with compositions intermediate between the stoichiometric ones as it was done in the case of potassium and barium borate systems. The concentration dependence of *K*_cr_ for the sodium borate glasses can be considered as reliable over the composition range from 0 to 20 mol.% Na_2_O. At the same time, there is no doubt that a more detailed study will reveal deviations from the interpolation curves shown in Figure 9 over the range of compositions from 20 to 40 mol.% Na_2_O.

The concentration dependences of *K*_cr_ for monolithic glasses (Figure 11 and Figure 13) show that, near each of the eutectic, the crystallization ability of glasses is suppressed. In the vicinity of many eutectics at low temperatures, volume crystallizing compounds form, such as 2K5B, K3B and 4Ba7B. According to modern concepts of the chemical structure of melts [29], each crystalline compound introduces its specific group into a melt. If one or two types of groups prevail in the melt, their clusters become centers of the crystallization. If three or four different types of groups are present in comparable quantities in the melt, it loses its ability to crystallize rapidly. None of the groups have enough material in their environment to form a critical nucleus. Such a melt can be called anti-crystalline. When the composition of the melt is close to the composition of one of the eutectic compounds, the structural groups of this compound begin to predominate quantitatively, and crystallization of this compound becomes possible. If the reason for the low crystallization of monolithic glasses of eutectic compositions is described correctly, we should expect that an unknown crystalline phase also forms near the BaB-30 eutectic.

## 5. Conclusions

The crystallization of the glasses of three borate systems, Na_2_O-B_2_O_3_, K_2_O-B_2_O_3_, and BaO-B_2_O_3_, was studied by DTA and XRPD. The specifics of the presented work are based on two essential points. Firstly, not only powders, but also massive monolithic glasses were systematically investigated. The DTA study of crystallization of massive monolithic glass samples makes it possible to obtain the structural information which is not distorted by the influence of the surface. Changes in the crystallization ability of monolithic glasses give information about changes in the structure of the glass itself. It was also shown that in the narrow vicinity of the eutectic compositions in the monolithic glasses there are regions with an anti-crystalline structure, incapable of the rapid crystallization. Secondly, the properly defined coefficient of the crystallization ability, *K*_cr_, of the glasses gives not only the possibility of a wide quantitative comparison both within the limits of one system, and different systems between themselves. The coefficient also made it possible to present subtle changes in the crystallization ability of monolithic glasses near eutectics in a clear and compact form.

## Figures and Tables

**Table 1:** The results of statistical processing of eight heating curves for NB-20 glass.

Thermal Parameters	Designation	Mean *T*, °С	Standard Deviation,°С	Relative Error, %
Onset of glass transition	*T*_g_	471	±1.6	0.2
Final of glass transition	*T*_g_’	500	±2.3	0.3
Onset of crystallization	*T*_x_	618	±3.8	0.4
Onset of melting	*T*_m_	806	±1.5	0.1
